# Inconsistencies in quality of life data collection in clinical trials: a potential source of bias? Interviews with research nurses and trialists

**DOI:** 10.1186/1745-6215-14-S1-O56

**Published:** 2013-11-29

**Authors:** Derek Kyte, Jonathan Ives, Heather Draper, Thomas Keeley, Melanie Calvert

**Affiliations:** 1 University of Birmingham, Birmingham, UK

## Aims

To explore reported inconsistencies in health related quality of life (HRQL) data collection in clinical trials.

## Methods

Semi-structured interviews were conducted with research nurses, data managers and trial coordinators involved in the collection of HRQL data in clinical trials. Recruitment took place across five sites in the UK: one primary care and two secondary care NHS trusts, and two clinical trials units. We used conventional content analysis, including methods of constant comparison and deviant case analysis, to analyze and interpret our data. Several processes were employed to ensure rigor, including regular team meetings aimed at facilitating reflexivity, member checking of interview summaries, peer review of verbatim interview transcripts and formal triangulation of coding.

## Results

26 individuals were interviewed. Participants reported: (1) inconsistent standards in HRQL measurement, both between, and within, trials, which appeared to risk the introduction of bias, (2) difficulties in dealing with HRQL data that raised concern for the well-being of the trial participant, which in some instances led to the delivery of non-protocol driven co-interventions, (3) a frequent lack of HRQL protocol content and appropriate training of trial staff, and (4) that HRQL data collection could be associated with emotional and/or ethical burden.

## Conclusions

Our findings suggest there are inconsistencies in the standards of HRQL data collection in some trials resulting from a general lack of HRQL-specific protocol content and training. These inconsistencies could lead to biased HRQL trial results. Future research should aim to develop HRQL guidelines and training aimed at supporting researchers to carry out high quality data collection.

